# Frameworks used to evaluate community-based rehabilitation interventions: A scoping review

**DOI:** 10.4102/ajod.v14i0.1546

**Published:** 2025-07-30

**Authors:** Sarah M. Manig, Liezel Ennion, Michael Rowe, Luc de Witte

**Affiliations:** 1 Department of Physiotherapy, Faculty of Community Health Science, University of the Western Cape, Cape Town, South Africa; 2 School of Health and Social Care, University of Lincoln, Lincoln, United Kingdom; 3 Research group Technology for Healthcare, Centre of Expertise Health Innovation, The Hague University of Applied Sciences, The Hague, The Netherlands

**Keywords:** community-based rehabilitation, evaluation, framework, scoping review, theoretical framework, impact assessment, rehabilitation evaluation

## Abstract

**Background:**

Community-based rehabilitation (CBR) interventions are important for improving the well-being of people with disabilities. However, there is no universally accepted framework for evaluating these interventions, which limits their effectiveness and integration into policy.

**Objectives:**

To explore theoretical frameworks used in evaluating CBR interventions, assessing their suitability, context-specific applicability and cultural relevance.

**Method:**

A scoping review methodology was employed to examine the literature. Databases searched included PubMed, CINAHL, EBSCOhost and Web of Science. Broad search terms and keywords used were CBR, analytical and/or methodological and/or theoretical and/or conceptual and/or evaluation framework, impact and evaluation. Only full-text articles written in English and published between 2000 and 2020 were included. Data were analysed using a narrative synthesis method.

**Results:**

No single framework has been widely recognised as the superior or most effective standard for evaluating CBR interventions. Instead, a combination of the CBR matrix and CBR guidelines was frequently used and adapted to be context-specific.

**Conclusion:**

While cultural relevance and context specificity are recognised as essential to the evaluation process – and measuring outcomes at the individual level is viewed as most appropriate – there remains a need for a certain level of standardisation.

**Contribution:**

The study highlights the need for context-specific and culturally relevant evaluation frameworks for CBR interventions, including appropriate outcome measures and/or evaluation instruments.

## Introduction

### Rationale

Community-based rehabilitation (CBR) has been widely adopted as a strategy to enhance the quality of life for people living with disabilities (PWD) by integrating health, education, social inclusion, empowerment and livelihood (Blose et al. 2021). The CBR matrix, which outlines these five pillars, provides a robust framework for guiding interventions. However, the application of this matrix in evaluating CBR interventions remains a challenge, as PWD continue to face entrenched issues such as poverty and human rights violations (Blose et al. 2021). While the matrix offers a comprehensive approach, its implementation must be flexible enough to adapt to different contexts, as the relative importance of each pillar may vary depending on the specific goals of the intervention and the population served.

Despite the advancement of the CBR matrix to guide CBR interventions to provide a comprehensive service, PWD remain in a poverty cycle and experience a gross infringement on their human rights (Blose et al. 2021). Limitations exist in evaluating CBR, as no consensus has been reached on the most appropriate tools or generic outcome measures when evaluating CBR interventions. Appropriate evaluation processes allow services to improve by exposing strengths and weaknesses.

The World Health Organization (WHO) has long defined ‘disability’ as an umbrella term encompassing impairments, activity limitations and participation restrictions (WHO 2011), which arise from the interaction between a person with a health condition and environmental and personal factors. Over a billion people are estimated to live with some form of disability, and the rates of disability continue to increase, partly because of ageing populations and the rising prevalence of chronic health conditions (WHO 2019). Historically, disability has been viewed through a deficit-based lens; however, in 2023, the WHO adopted the concept of Positive Health, emphasising well-being as a dynamic ability to adapt and manage life’s challenges (Van Vliet et al. 2024). This shift significantly impacts the way disability prevalence is understood and counted, as it expands the scope beyond mere impairments to include an individual’s capacity to thrive within their context. Community-based rehabilitation aligns well with this framework, as it promotes social inclusion, empowerment and functional ability rather than solely focusing on medical limitations. Recognising disability within this broader construct reinforces the need for robust, context-specific CBR methodologies that support both individuals and their communities (Mason et al. 2017).

In low-middle-income countries (LMICs), PWD are often unable to contribute to the household income and are dependent on family members for daily tasks. A lack of independence for PWD has a negative impact on the national economy and health care structures as there is a growing demand for government financial aid and health resources.

Global rural population has reached 3.4 billion with close to 90% of the world’s rural population living in Africa and Asia (International Labor Office 2018). The rural population of the world has grown slowly since 1950 and is expected to reach 3.1 billion by 2050 (International Labor Office 2018). Already, access to health care is very challenging for persons living in rural areas, and a predicted increase in population size will place further strain on existing social services.

The global population size is on the increase, and with it, the number of PWD (International Labor Office 2018). People living with disabilities face various barriers within their communities, particularly those related to accessing medical services, especially in low-income or rural areas. Community-based rehabilitation is the most effective approach for improving the well-being of PWD and fostering increased community participation in these settings. However, there is currently no consensus or framework for evaluating the impact of CBR interventions, limiting their uptake into policy (Thomas & Thomas 1999).

Accessing medical services is vital for PWD to promote health, prevent deterioration, improve overall function and community participation and strive for the optimal quality of life (WHO 2010). A consensus evaluation framework can contribute to the effectiveness and sustainability of CBR interventions, ultimately improving the well-being and participation of those living with disabilities, particularly in rural and low-income areas.

There is a sense of urgency for the development of research and, more specifically, a framework that stands to evaluate interventions. Boyce and Ballantyne (2000) argue that evaluating CBR interventions is often not a priority for project leaders but emphasise that CBR cannot be sustained without appropriate evaluation mechanisms to assess its foundational information base. Furthermore, they believe that evaluation, if appropriately conducted, can help the development of community programmes. Quantifiable evidence is essential for informing policy decisions. Without it, there is no objective basis for advocating national or international change at the governmental level.

### Research questions

The following research question guided the review:

‘*What are the existing frameworks or models that have been used to evaluate CBR interventions*?’ and ‘*How usable and relevant are these frameworks to the context of CBR*?’

### Objective

The objective was to explore theoretical frameworks used in evaluating CBR interventions, assessing their suitability, context-specific applicability and cultural relevance.

## Methods

A scoping review was conducted, and the methodology is detailed in the following section.

### Search strategy

#### Eligibility criteria

**Inclusion criteria:** Only full-text publications published between 2000 and 2020 in English were included in the search. All study designs were included.

**Exclusion criteria:** Grey literature, non-peer reviewed publications.

The search terms were used in different combinations using Boolean phrases to maintain a broad search and not exclude relevant titles (see Table 1):

- ‘Community-based Rehabilitation and/or framework and/or impact’
- ‘Community-based Rehabilitation and/or framework and/or evaluation’
- ‘Community-Based Rehabilitation and/or evaluation framework and/or impact’
- ‘Community-based Rehabilitation and/or framework and/or efficacy’
- ‘Community-based Rehabilitation and/or conceptual framework and/or impact’
- ‘Community-based Rehabilitation and or theoretical framework and/or impact’
- ‘Community-based Rehabilitation and/or methodological framework and/or impact’.

**TABLE 1:** Search terms.

Community based rehabilitation (CBR)	
Framework	Impact, evaluation or efficacy
Evaluation framework	-
Conceptual framework	Impact
Theoretical framework	Impact
Methodological framework	Impact

#### Information sources

The first search commenced on 01 September 2020. The following databases were included in the search: PubMed, Ebsco-Host, CINAHL and Web of Science (*n* = 224).

**Study process:** After the removal of duplicates (*n* = 145), the references were imported into Microsoft Excel to be screened. The following *inclusion criteria* had to be met for the research to qualify as relevant:

- Titles and abstracts relevant to the research question.
- Intervention of the study falls within the CBR matrix.
- Intervention uses an evaluation model or framework.

Two reviewers screened the titles for inclusion.

### Data collection process

#### Study selection

After the initial screening of the titles, 37 articles met the inclusion criteria and were included in the next step. The abstracts of these relevant titles were screened using the inclusion criteria described above. Agreement between the reviewers was good (Kappa = 0.81). Upon further inspections, articles found to be relevant were reviewed in full text by the first author. Publications that did not meet the inclusion criteria were excluded.

**FIGURE 1:**
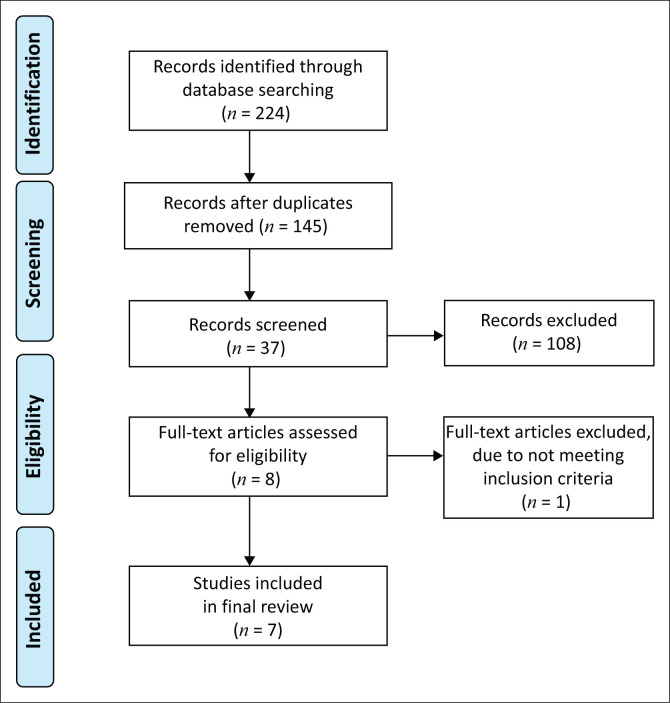
Literature search and study selection.

A total of 224 titles were retrieved across all databases in the initial title search. After the removal of duplicate titles, 145 unique titles remained. Of these, 37 were deemed relevant to the research question and moved into the second stage in which the abstracts were read for relevance. Finally, seven full-text articles were aligned with the research question, met the inclusion criteria and were included in the scoping review (see Figure 1).

#### Synthesis methods

After the final full-text inclusion, a data extraction form was developed in alignment with the research question. This data extraction form consisted of descriptive elements (see Table 2) which included the author, year, title, aim, study design, instruments of data collection, study population, frameworks identified, areas of the CBR matrix the study engaged with and the level (individual, organisational and contextual) at which the intervention was evaluated. The relevant data were extracted from the articles and integrated into the form.

**TABLE 2:** Table of study characteristics.

Author, date, title	Aim	Design	Setting and/or population
Adeoye, Seeley & Hartley (2011). Developing a tool for evaluating community-based care in Uganda.	Develop a prototype tool for evaluation of a CBR programme	Case study	Tororo, Uganda Young adults with disabilities
Chung, Packer and Yau (2011). A framework for evaluating community-based rehabilitation programmes in Chinese communities.	Develop a framework to assess the quality of Chinese CBR programme	Case study	China Children with disabilities
Biggerri and Ferrannini (2014). Opportunity gap analysis: Procedures and methods for applying the capability approach in development initiatives.	Analysing complex programming using the capability approach framework and to present an innovative procedure for the systematised assessments of capabilities (opportunity freedom) in development projects	Case study	West-Nile region, Uganda. Fourteen young adults (18–24 years old)
Grandisson et al. (2016). Expert consensus on best evaluative practices in community-based rehabilitation.	Generate expert consensus on best evaluative practices for CBR	Delphi study	Online (participants recruited globally) 61 experts
Luruli, Netshandama and Francis (2016). An improved model for provision of community-based health care rehabilitation services in Vhembe District, Limpopo Province of South Africa.	Suggest an improvement in the model of providing CBR services	Case study Mixed methods with an exploratory and descriptive nature	Vhembe District in Limpopo Province of South Africa. 2850 people who have disabilities from the CBR programme
Scobbie et al. (2013). Implementing a framework for goal setting in community-based stroke rehabilitation: A process evaluation.	Understand or explore the implementation, acceptability and perceived benefits of the G-AP framework	Case study	United Kingdom; eight patients who suffered a CVA
Velema and Cornielje (2003). Reflect before you act: Providing structure to the evaluation of rehabilitation programmes.	Evaluating potentially diverse rehabilitation programmes	Meta-analysis	-

Note: Please see the full reference list of the article, Manig, S.M., Ennion, L., Rowe, M. & De Witte, L., 2025, ‘Frameworks used to evaluate community-based rehabilitation interventions: A scoping review’, African Journal of Disability 14(0), a1546. https://doi.org/10.4102/ajod.v14i0.1546

CBR, community-based rehabilitation; CVA, cerebral vascular accident; PWD, people with disabilities.

Initially, the overall data were examined to determine the study aim, design, data collection methods, population and setting. Thereafter, the key findings of the sources were collated based on the relevance to the study aim and research question, which included frameworks identified, CBR matrix, and the level at which the study was evaluated (individual, context and/or programme).

### Ethical considerations

This scoping review was conducted in accordance with the ethical standards outlined by the University of the Western Cape’s Biomedical Research Ethics Committee. Permission to conduct this research was obtained from the Biomedical Research Ethics Committee (Reference number: BM20/5/31).

## Review findings

### Study characteristics

Most of the publications used a case study approach to conduct their study in low-income communities in Africa and Asia (Adeoye, Seeley & Hartley 2011; Biggerri & Ferrannini 2014; Chung et al. 2011; Luruli et al. 2016). The remaining publications either represented an online community or a high-income country, such as the United Kingdom (Scobbie et al. 2013). The case studies were mostly focused on the framework or concept in question and how it interacted with the setting and community.

The studies by Grandisson et al. (2016) and Velema and Cornielje (2003) had a broader focus on available literature and creating concepts and suggested frameworks to be used in a CBR context (see Table 2).

### Frameworks identified

None of the studies identified a single framework applicable to all CBR interventions (Table 3). This likely reflects the diverse and context-specific nature of CBR interventions, which necessitate tailored evaluation frameworks. Instead, most of the case studies utilised a combination of established white paper literature (such as the CBR matrix and CBR guidelines) and the institution’s own documentation to create a unique, context-specific framework for evaluating the specific programmes under investigation.

**TABLE 3:** Results of individual included studies.

Author, date, title	Framework	CBR pillars	Evaluation
Adeoye, Seeley & Hartley (2011). Developing a tool for evaluating community-based care in Uganda.	Joint position statement	All	Organisational level
Chung et al. (2011). A framework for evaluating community-based rehabilitation programmes in Chinese communities.	Final framework: Five domains, 25 categorised core elements and 72 indicators.	All	Contextual and individual
Biggerri and Ferrannini (2014). Opportunity gap analysis: Procedures and methods for applying the capability approach in development initiatives.	Dynamic analytical framework: Identifies opportunity gaps between the community and individual	Social Participation Livelihood	Contextual: Project and frameworks looked at a collective (group of people who have disabilities) perceptions of social inclusion and/or participation and empowerment.
Grandisson et al. (2016). Expert consensus on best evaluative practices in community-based rehabilitation.	CBR matrix and CBR principles	Social Community Education Empowerment	Contextual: Delhi study looking at best evaluative practice in CBR
Luruli et al. (2016). An improved model for provision of community-based health care rehabilitation services in Vhembe District, Limpopo Province of South Africa.	Adapted conceptual model for the client satisfaction evaluation using SERVQUAL† model	Health Education Social Livelihood	Individual Contextual Organisational
Scobbie et al. (2013). Implementing a framework for goal setting in community-based stroke rehabilitation: A process evaluation.	G-AP	Health Lifestyle Social	Individual Contextual
Velema and Cornielje (2003). Reflect before you act: Providing structure to the evaluation of rehabilitation programmes.	Sequential question framework	All	Individual Contextual Organisational

Note: The G-AP Framework has been developed to support person-centred goal setting practice; Please see the full reference list of the article, Manig, S.M., Ennion, L., Rowe, M. & De Witte, L., 2025, ‘Frameworks used to evaluate community-based rehabilitation interventions: A scoping review’, African Journal of Disability 14(0), a1546. https://doi.org/10.4102/ajod.v14i0.1546

SERVQUAL, Service quality framework; G-AP, The Goal Setting and Action Planning; CBR, Community-based rehabilitation.

† , see Parasuraman et al. 1988.

Only one study deviated from this approach, focusing on an existing evaluation method known as the Goal Setting and Action Planning (G-AP) framework (Scobbie et al. 2013). The G-AP framework is designed to evaluate complex interventions through goal setting and action-planning principles. It employs action plans and coping strategies to achieve outlined goals and overcome anticipated barriers. An intervention’s success is measured by the achievement of these goals.

Velema and Cornielje (2003) advocate for a reflective approach before commencing evaluations. They emphasise the importance of a solid foundation where systematic information is gathered about the individual, services, programme, programme environment and the interrelationships between these elements. Their study introduced a sequential question framework designed to stimulate critical thinking and define the evaluation’s purpose before it begins.

The Delphi study conducted by Grandisson et al. (2016) involved 42 experts in CBR to determine the best evaluative practices. The consensus among experts was that the most suitable framework for evaluation would be a combination of the CBR matrix and the guiding principles of CBR. The major pillars of the CBR matrix – Health, Education, Social, Livelihood and Empowerment – provide a structured framework for evaluations, ensuring they remain context-specific and culturally respectful.

## Implications and recommendation

This study reviewed the available literature on frameworks used to evaluate CBR interventions globally over the past 20 years, with the most recent study included published by Grandisson et al. in 2016. The seven articles included in this review do not represent the entirety of information on frameworks for CBR evaluation. However, they indicate a limited amount of published scientific literature on the topic. There is a significant need for more research and published articles on evaluating CBR interventions.

A notable publication bias exists in this area, potentially excluding valuable information. Specifically, many unpublished works, non-English publications and grey literature may provide insights into CBR evaluation frameworks that are not captured in this review. Including these sources could offer a more comprehensive understanding and reveal effective frameworks that are not widely recognised.

There is no universally applicable framework for evaluating CBR interventions (Grandisson et al. 2016; Velema & Cornielje 2003). However, it is generally agreed that the most suitable framework would be a combination of the CBR matrix and/or CBR guidelines and an approach that ensures context specificity and cultural relevance. Community-based rehabilitation is a diverse and holistic practice that operates in various contexts. Thus, the approach to monitoring and evaluating CBR interventions must anticipate and accommodate this diversity.

The authors agree that tailoring the framework to be context-specific is essential for its relevance and effectiveness. It is also agreed that outcomes are best evaluated at an individual level to measure the intervention’s effectiveness (Adeoye, Seeley & Hartley 2011; Biggerri & Ferrannini 2014; Chung et al. 2011). This is critical in the standardisation of a framework. Tailoring the evaluation framework to a specific context or environment allows the use of appropriate outcome measures at an individual level, providing a more precise and accurate evaluation at a programme level.

The authors in this review recommend using the major pillars of the CBR matrix (and their subheadings) to guide the format of an evaluation for a CBR intervention. The main categories of the CBR matrix – Health, Education, Social, Livelihood and Empowerment – provide structure to the direction and area being evaluated. This structure should then be made context specific using programme documents and stakeholder consultations (Adeoye, Seeley & Hartley 2011; Biggerri & Ferrannini 2014; Chung et al. 2011; Luruli et al. 2016; Mason et al. 2017).

### Limitations

Although this review attempts to uncover literature on frameworks used to evaluate CBR interventions, several limitations are acknowledged. The date range of published literature was restricted to 2000–2020, excluding research done before or after this period. The search only included English publications, excluding potentially relevant studies from non-English speaking low-income and middle-income countries where CBR is more commonly implemented. Only full-text articles published in peer-reviewed journals were included, omitting grey literature that could provide additional insights.

## Conclusion

This scoping review did not identify a single framework that experts universally regard as the gold standard. Instead, it revealed expert consensus favouring the combined use of theoretical frameworks, such as the CBR matrix and CBR guidelines, to tailor evaluations to be context specific and culturally relevant. While cultural relevance and context specificity are recognised as essential to the evaluation process – and measuring outcomes at the individual level is viewed as most appropriate – there remains a need for a certain level of standardisation. Specifically, standardisation of core outcome measures or evaluation instruments would enhance comparability and enable shared learning across different CBR programmes without compromising necessary contextual flexibility. Further research is needed to better understand how evaluation frameworks evolve and to identify current instruments or outcome measures that could inform the development of standardised yet adaptable evaluation practices in CBR interventions.
